# Supplementary material for: Barriers and Expectations of Adolescents Regarding the Identification and Management of Their Psychoactive Substance Use by Their General Practitioner
Source: Int J Environ Res Public Health. 2022 Oct 14;19(20):13231. doi: 10.3390/ijerph192013231 (PMC9603752; doi:10.3390/ijerph192013231)
Supplement: Supplementary file 1 [file ijerph-19-13231-s001.zip › ijerph-1941284-supplementary.pdf]

This survey is only for adolescents ***aged 12-17 years****.*

As part of our study, we are interested in your opinion about the accessibility of your doctor to talk about your use of alcohol, tobacco, cannabis, and other drugs.

All you must do is answer this ***ANONYMOUS*** *questionnaire (neither your doctor nor your parents will have access to the results). It will only take you 10 minutes.*

Thank you for your time.

1) Why are you coming to see your doctor today?

…………………………………………………………………………

2) How many times have you visited your doctor in the last 12 months?

.... times

Here are some questions about your use of alcohol, tobacco, cannabis, and other drugs

3) In the past 12 months, have you used any of these products, and if so, how often? (Check only one answer per line)

	Never	Occasionally	About once a month	On weekends or once or twice a week	≥3 times a week but not every day	Every day
Alcohol	☐	☐	☐	☐	☐	☐
Tobacco	☐	☐	☐	☐	☐	☐
Cannabis	☐	☐	☐	☐	☐	☐
Cocaine	☐	☐	☐	☐	☐	☐
Glue, Poppers, Solvents	☐	☐	☐	☐	☐	☐
LSD, ecstasy	☐	☐	☐	☐	☐	☐
Heroin, cough medicine	☐	☐	☐	☐	☐	☐
Amphetamines, Speed	☐	☐	☐	☐	☐	☐

If you have ***never*** *used any of the products mentioned in question 3, go directly to question 10.*

4) **At what age did you start using?** (Fill in only the boxes that apply to you; if you have never used the products listed below on a regular basis, i.e. at least once a week for at least one month, do not fill in the right-hand column of the table)

	For the first time	Regularly (at least once a week for at least 1 month)
Alcohol	………. years	………. years
Tobacco	………. years	………. years
Cannabis	………. years	………. years
Other drugs	………. years	………. years

5) **Have you ever injected drugs in your life?**

☐ Yes ☐ No

6) **Have you used alcohol or other drugs in the past 30 days?**

☐ Yes ☐ No

7) **a) In the past 12 months, how often have you had 5 or more drinks on one occasion?**

……………………………………………………. times

b) If you are a boy, how many times have you had 8 or more drinks on one occasion?

……………………………………………………. times

8) **In the past 12 months, has this happened to you?** (Check one box per line)

	Yes	No
Your alcohol or drug use has affected your physical health (digestive problems, infections, injuries...)	☐	☐
You have had psychological difficulties because of your alcohol or drug use (anxiety, depression, concentration problems, suicidal thoughts...)	☐	☐
Your drinking or drug use has affected your relationship with your family	☐	☐
Your drinking or drug use has harmed a friendship or romantic relationship	☐	☐
You have had difficulties at school because of your alcohol or drug use (absence, exclusion, drop in grades...)	☐	☐
You have spent too much money or lost a lot of money because of your drinking or drug use	☐	☐
You committed a delinquent act while under the influence of alcohol or drugs, even if the police did not arrest you (theft, selling drugs, driving...)	☐	☐

You took risks while using alcohol or drugs (unprotected sex, playing sports after drinking or using drugs...)	☐	☐
You felt that the same amount of alcohol or drugs now had less effect on you	☐	☐
You have talked to someone (doctor, school nurse, psychologist...) about your alcohol or drug use	☐	☐

The expectations you have of your doctor

9) Have you ever talked to your doctor about your drinking?

	Yes	No
Alcohol	☐	☐
Tobacco	☐	☐
Cannabis	☐	☐
Other drugs	☐	☐

10) Has your doctor ever asked you about your use?

	Yes	No
Alcohol	☐	☐
Tobacco	☐	☐
Cannabis	☐	☐
Other drugs	☐	☐

11) Would you like to talk to her about it?

☐ Yes ☐ No

a) **If yes, why?** (You can check more than one box)

☐ You trust him
☐ You don't feel judged
☐ You feel comfortable
☐ He listens to you
☐ This is its role
☐ He takes his time
☐ You know he's not going to tell your parents
☐ His questions are appropriate
☐ You know you're not going to disappoint him
☐ He understands you
☐ It is competent to
☐ Other: …………………………………………………

b) If not, why not? (You can check several boxes)

☐ You don't trust him
☐ You're afraid he'll tell your parents
☐ You feel judged
☐ His questions are inappropriate
☐ You don't feel comfortable
☐ You are afraid of disappointing him
☐ He doesn't listen to you
☐ You are afraid of disappointing him
☐ This is not its role
☐ He is incompetent
☐ He doesn't take his time
☐ Other: ………………………………………………….

12) Is there anyone you could talk to about this? (You can check more than one box)

☐ Your parents
☐ Another member of your family
☐ Your friends
☐ The school nurse
☐ The pharmacist
☐ An association

13) Would a questionnaire, to be filled out during a consultation, help you discuss these topics with your doctor?

☐ Yes ☐ No

14) Would you be willing to talk honestly about your substance use in front of your parents during a consultation?

☐ Yes ☐ No

15) Concerning cannabis, does the fact that it is illegal make it difficult for you to talk about it with your doctor?

☐ Yes ☐ No

16) Has your doctor ever talked to you?

	Yes	No
Young consommer consultations	☐	☐
Addiction care, support and prevention centers	☐	☐
Reception and support centers for harm reduction for drug users	☐	☐
From the « teenager's house »	☐	☐
Telephone platforms or websites (drogue Info...)	☐	☐
Other: ………………………………………………………………………………………….		

Finally, a few questions about you

17) Are you:

☐ a Boy ☐ a Girl

18) How old are you?

.... years old

19) How do you live?

☐ Alone
☐ With at least one of your parents
☐ With another member of your family
☐ In a host family
☐ In a foster home
☐ With friends or your boyfriend or girlfriend
☐ Other: …………………………………………………

20) What is your postal code?

....

21) What is your situation?

☐ Active
☐ Student in the middle school
☐ Student in general high school
☐ Student in technological high school
☐ Student in vocational or agricultural high school
☐ Student in an apprentice training center or in a rural family home
☐ Other: …………………………………………………

Thank you for completing this survey. You can fold it and put it in the ballot box.

If you wish, do not hesitate to talk to your doctor about these subjects.
